# Postembryonic development of transit amplifying neuroblast lineages in the *Drosophila *brain

**DOI:** 10.1186/1749-8104-4-44

**Published:** 2009-12-11

**Authors:** Natalya Izergina, Jasmin Balmer, Bruno Bello, Heinrich Reichert

**Affiliations:** 1 Biozentrum, University of Basel, CH-4056 Basel, Switzerland

## Abstract

**Background:**

Specific dorsomedial (DM) neuroblast lineages of the *Drosophila *brain amplify their proliferation through generation of transit amplifying intermediate progenitor cells. Together, these DM neuroblast lineages comprise over 5,000 adult-specific neural cells and thus represent a substantial part of the brain. However, no information is currently available about the structure or function of any of the neural cells in these DM lineages. In this report we use MARCM-based clonal analysis together with immunocytochemical labeling techniques to investigate the type and fate of neural cells generated in the DM lineages.

**Results:**

Genetic cell lineage-tracing and immunocytochemical marker analysis reveal that DM neuroblasts are multipotent progenitors that produce a set of postembryonic brain glia as well as a large number of adult-specific protocerebral neurons. During larval development the adult-specific neurons of each DM lineage form several spatially separated axonal fascicles, some of which project along larval brain commissural structures that are primordia of midline neuropile. By taking advantage of a specific Gal4 reporter line, the DM-derived neuronal cells can be identified and followed into early pupal stages. During pupal development the neurons of the DM lineages arborize in many parts of the brain and contribute to neuropile substructures of the developing central complex, such as the fan-shaped body, noduli and protocerebral bridge.

**Conclusions:**

Our findings provide cellular and molecular evidence for the fact that DM neuroblasts are multipotent progenitors; thus, they represent the first identified progenitor cells in the fly brain that have neuroglioblast functions during postembryonic development. Moreover, our results demonstrate that the adult-specific neurons of the DM lineages arborize widely in the brain and also make a major contribution to the developing central complex. These findings suggest that the amplification of proliferation that characterizes DM lineages may be an important requirement for generating the large number of neurons required in highly complex neuropile structures such as the central complex in the *Drosophila *brain.

## Background

The *Drosophila *brain is a highly complex structure composed of tens of thousands of neurons that are interconnected in numerous exquisitely organized neuropile structures, such as the mushroom bodies, antennal lobes and central complex. The neurons of the central brain, defined as the supraesophageal ganglion without the optic lobes, derive from approximately 100 bilaterally symmetrical pairs of neural stem cell-like neuroblasts, each of which is thought to generate a characteristic lineage of neural progeny [1,2]. Several studies have indicated that each developing neuroblast acquires an intrinsic capacity for neuronal proliferation in a cell-autonomous manner and generates a specific lineage of neural progeny that is nearly invariant and unique. This implies that each neuroblast acquires a specific identity that determines the number and types of neural progeny it generates. This specification of neuroblasts has been shown to occur through a combination of positional information, and temporal and combinatorial cues provided by the suite of developmental control genes expressed by each precursor (for reviews, see [3-5]).

Neuroblasts begin to proliferate during embryonic development and during this initial phase of proliferation they generate the primary neurons of the larval brain. After a period of mitotic quiescence during the early larval period, most brain neuroblasts reactivate proliferation and produce secondary neurons that make up the bulk of the adult brain; these are referred to as adult-specific neurons [6,7]. Indeed, 95% of the neurons in the adult brain are secondary neurons generated during postembryonic development. These adult-specific neurons initially form a lineage-related cluster of immature neurons that extend fasciculated primary neurites into the neuropile but wait until metamorphosis to complete their extension to synaptic targets and final morphogenesis [8-11].

Most neuroblasts in the central brain generate lineages comprising, on average, 100 to 120 adult-specific cells [12]. (The neuroblasts that generate the intrinsic cells of the mushroom bodies each produce an average of approximately 200 adult-specific cells; these neuroblasts do not enter a quiescent state in early larva.) In contrast, remarkably large neuroblast lineages are generated in the dorsomedial (DM) area of the larval brain. The number of adult-specific cells in these DM neuroblast lineages averages 450, more than twice the average number of cells in the mushroom body lineages [12]. The large number of neurons in these lineages is achieved by an amplification of neuroblast proliferation through generation of intermediate progenitor cells. Most neuroblasts in the central brain divide asymmetrically in a stem cell mode whereby they self-renew and generate smaller daughter cells called ganglion mother cells, which divide once to produce two postmitotic progeny [4,5,13-15]. In contrast, dividing DM neuroblasts (also referred to as posterior asense-negative (PAN) neuroblasts or type II neuroblasts) self-renew and generate intermediate progenitor cells that act as transit amplifying cells and can generate numerous ganglion mother cell-like cells by retaining their ability to divide several more times [12,16,17]. In this respect, neurogenesis in DM lineages is similar to that seen in the mammalian central nervous system in which the primary progenitors amplify the progeny they produce through the generation of proliferating intermediate progenitors [18,19]. (In addition to the six pairs of DM neuroblasts located in the dorsomedial area of the brain, there are two additional pairs of PAN (type II) neuroblasts located more laterally in the brain [17]; because they are easier to identify, we focused our analysis on the six DM neuroblasts.)

The six bilaterally symmetrical pairs of DM (type II) neuroblast lineages together generate over 5,000 adult-specific cells due to the amplification of neuroblast proliferation [12]. Given current estimates of total cell number in the *Drosophila *brain [20], this cell number would roughly correspond to one-fourth of the total number of cells in the central brain. The DM lineages thus represent a substantial part of the brain. However, no information is currently available about the phenotypic fate of any of the neural cells in the DM lineages. It is not known if the cells in these lineages are exclusively neuronal or if glial cells are also generated. Nor is it known if the neurons in these lineages are involved in the formation of specific complex neuropile structures or if they project widely throughout the brain. This total lack of information on the type of cells generated and their roles in brain circuitry thus represents a major obstacle in understanding the development of the fly brain.

## Results

### DM neuroblast lineages contain adult-specific neurons and glial cells

During larval development, six DM neuroblasts generate large lineages, each of which consists of an average of 450 cells that are located at the DM midline of each hemisphere [12,17]. These DM lineages can be identified based on their overall size and position by using mosaic-based MARCM (mosaic analysis with a repressible cell marker) techniques to label neuroblasts and their postembryonic progeny in the larval brain. For this, random mitotic recombination was induced in neuroblasts by heat-shock induction of FLP within a few hours after larval hatching in order to obtain positive labeling of the neuroblasts and their clonal post-mitotic progeny (hereafter referred to as neuroblast clones). Green fluorescent protein (GFP)-labeled neuroblast clones corresponding to each of the six DM lineages were recovered at the late third instar stage and co-stained with neural cell-specific molecular labels. All of these clones consisted of more than 350 cells and were thus significantly larger than any of the other neuroblast lineages in the larval brain [12]. Clonal cell numbers were not significantly different if MARCM labeling was induced in the larva a few hours after hatching (450 cells; range 370 to 580) or in the embryo at stage 13 (468 cells; range 362 to 545), underscoring the fact that most neurons in the fly brain are generated postembryonically.

**Figure 1:**
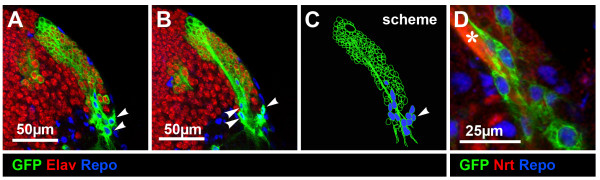
**DM neuroblast lineages contain both neurons and glial cells**. **(A, B) **Consecutive confocal images (from dorsal to ventral) of a DM MARCM clone (GFP, green). Neuronal cells are Elav-positive and glial cells are Repo-positive (blue, arrowheads). **(C) **Scheme of the DM lineage: note that localization of the glial cells is distal to the neuroblast in the clone. **(D) **A close-up view of the DM glial cells. Secondary neuronal cells, their projections and common axonal bundle are stained with anti-Neurotactin (Nrt, red) and glial cells are Repo-positive (blue). Glial cells extend processes along the axonal bundle (asterisk).

All postembryonic DM lineages contained a set of intermediate neuronal progenitors located near the neuroblast; these cells have been described previously [12,16,17] and are not considered further here. To determine if the post-mitotic cells in the DM lineages were all adult-specific neuronal cells or if they also comprised glial cells, GFP-labeled neuroblast clones were co-labeled with the neuron-specific marker anti-Elav and the glial cell-specific marker anti-Repo. In all cases, we found that the great majority of the cells in DM lineages were Elav-positive and thus corresponded to neuronal cells; however, we also found that DM lineages consistently contained Repo-positive, Elav-negative glial cells (Figure 1A-C). These DM-derived glial cells were located distal to the neuroblast in the clone of labeled cells. The average number of GFP-labeled glial cells in DM neuroblast clones at 96 hours after larval hatching was 13 ± 4.4. DM clones recovered at 72 hours after larval hatching also contained an average of 13 ± 6.1 cells, suggesting that most of the glia had been generated by this time.

The DM lineage glial cells had the general structural features of neuropile glia. In the late third larval instar, their cell bodies were generally clustered in the vicinity of the emergent secondary axon tracts of the DM lineages, and their processes were associated with these tracts (Figure 1D). Furthermore, DM lineage glial processes were also often associated with larval interhemispheric commissures. Repo-positive surface and cortex glia located at the dorsal midline immediately adjacent to the DM clones were never GFP labeled. Since the DM lineages contain both neurons and glia, the DM neuroblasts are, in terms of their proliferative potential, neuroglioblast-like. They are likely to be the only neural progenitors with neuroglioblast features present in the postembryonic brain; all other postembryonically generated glia are thought to be generated by symmetrically dividing glial precursors [21]. In accordance with this, when we examined 153 non-DM (canonical) postembryonic neuroblast lineages we never found glial cells in the labeled clones.

Taken together, these findings uncover an unexpected feature of these neuroblast lineages; they are not only by far the largest in terms of overall cell number, they also comprise glial cells and thus represent the only known neuroglioblast lineages in the postembryonic central brain.

### DM neurons form commissural and longitudinal secondary axon tracts in the larval brain

To study the axonal projections of the adult-specific neurons of DM lineages in the larval brain, we recovered MARCM-labeled neuroblast clones corresponding to each of the six DM lineages and co-immunostained these with an anti-Neurotactin antibody that labeled secondary neurons and their axon tracts (clones were induced at early first instar stage and recovered at the late third instar). In all DM lineages, axons from the adult-specific neuronal cells fasciculated to form initial secondary axon tracts within the cortical layer. As soon as these initial secondary axon tracts reached the brain neuropile, they split into several subsidiary tracts. This contrasted with the behavior of secondary tracts in most other brain lineages, which generally formed a single, discrete fascicle of axons within the brain cortex and neuropile during larval stages [2,11]. In all six DM lineages, some of these subsidiary secondary tracts projected into the interhemispheric commissures while others formed ipsilateral descending or ascending projections. Similar to the secondary axon tracts formed by other neuroblast lineages in the late larval brain, these DM-derived secondary axon tracts traveled for variable distances within the brain hemispheres but had not yet evolved into the long axon tracts that characterize the adult brain.

The DM lineages formed a set of secondary axon tracts with a characteristic and relatively invariant trajectory in the larval brain neuropile. Based on the rostral-to-caudal arrangement of the cell body clusters of the six DM lineages in the larval brain hemispheres, we numbered each of these as follows: DM1 (most rostral), DM2, DM3, DM4, DM5, DM6. The characteristic neuroanatomical features of the secondary axon tracts formed by all six DM lineages in the late third instar larval brain are described below. For a given DM lineage, these anatomical features were the same irrespective of whether MARCM clones were induced in the stage 13 embryo or in the early larva.

**Figure 2:**
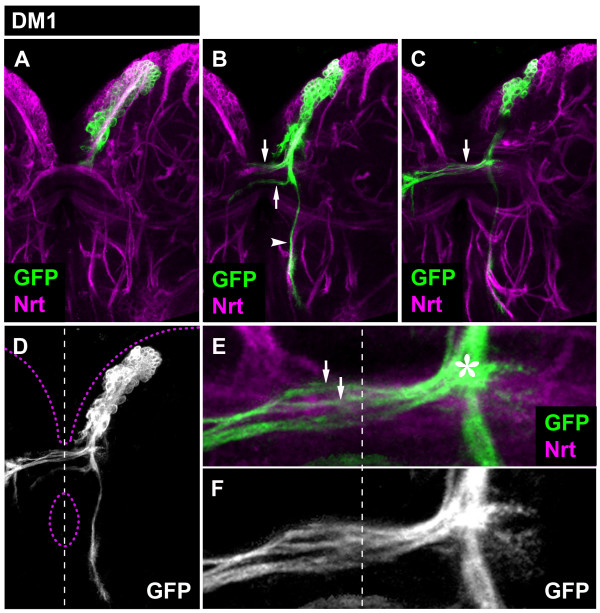
**Projection pattern of the larval DM1 lineage**. **(A-C) **Consecutive confocal images (from dorsal to ventral) of DM1 MARCM-labeled clone (GFP, green). Secondary neurons and their projections are stained with anti-Neurotactin (Nrt, magenta). Note commissural (arrows) and longitudinal (arrowheads) projections. **(D) **Z-projection of the entire DM1 lineage. Brain and oesophagus outline shown as a magenta dashed line; midline is a white dashed line. **(E, F) **A close-up view of the commissural projections. Note how the axonal bundle defasciculates (arrows) upon entering the commissure and that a subset of these axons enters the contralateral brain hemisphere. Also note that DM1 axons enter the commissure at a most medial site (asterisk).

The DM1 lineage formed a secondary axon tract that projected towards the interhemispheric region, where it separated into two fascicles: the first fascicle entered a larval interhemispheric commissure and immediately split into multiple commissural fiber bundles; the second fascicle formed a descending projection, from which an additional small commissural bundle also branched off (Figure 2A-D). The commissural fiber bundles crossed the midline and a subset of these entered the neuropile of the contralateral hemisphere; within the commissures these fiber bundles appeared to defasciculate into a network of smaller axon bundles that projected along multiple commissural pathways (Figure 2E, F). The larval commissure in which these fibers projected corresponds to the DPC1 commissure as defined by Pereanu and Hartenstein [11]. The descending fascicle, which in contrast remained tightly bundled, projected along the medial edge of the ipsilateral hemisphere.

**Figure 3:**
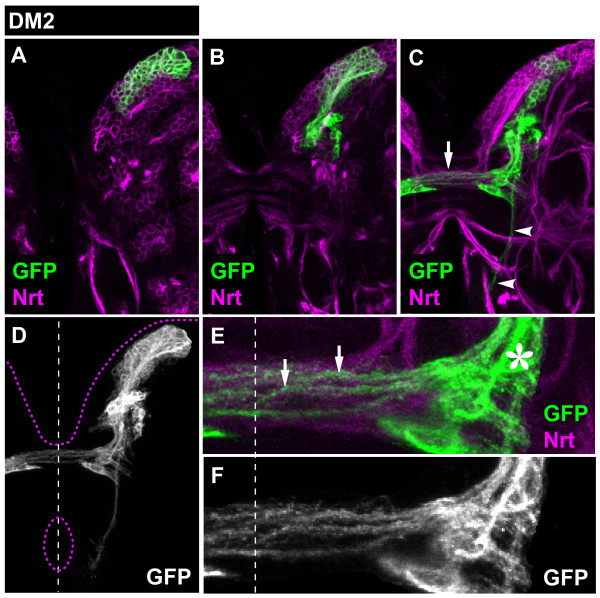
**Projection pattern of the larval DM2 lineage**. **(A-C) **Consecutive confocal images (from dorsal to ventral) of DM2 MARCM-labeled clone (GFP, green). Secondary neurons and their projections are stained with anti-Neurotactin (Nrt, magenta). Note commissural (arrows) and longitudinal (arrowheads) projections. **(D) **Z-projection of the entire DM2 lineage. Brain and oesophagus outline shown as a magenta dashed line; midline is a white dashed line. **(E, F) **A close-up view of the commissural projections. Note how the axonal bundle defasciculates (arrows) upon entering the commissure and that a subset of these axons enters the contralateral brain hemisphere. Also note the site where DM2 axons enter the commissure (asterisk).

The DM2 lineage formed a secondary axon tract that projected toward the commissural system and also split into multiple commissural fiber bundles and one descending fascicle (Figure 3A-D). The set of DM2 commissural bundles entered the same larval commissure as the DM1 commissural fascicle, but using a different site of entry from that used by the DM1 fibers. (We refer to these commissure entry sites as site 1 and site 2, respectively.) DM2-derived commissural bundles defasciculated into smaller lattice-like projections that crossed the midline, and some of these entered the contralateral hemisphere (Figure 3E, F). Glial cells from the DM2 lineage were often observed near the site of commissure entry. The single descending fascicle projected along a pathway that was roughly parallel to the one formed by the DM1 descending fascicle but was located more laterally in the hemisphere.

**Figure 4:**
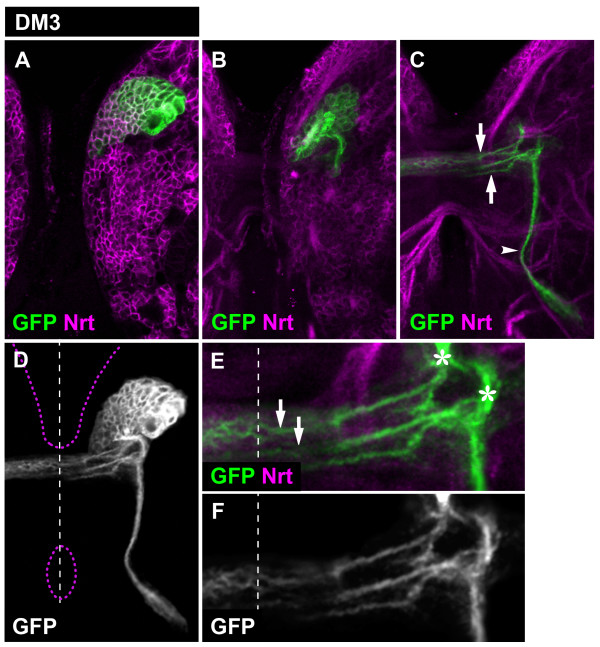
**Projection pattern of the larval DM3 lineage**. **(A-C) **Consecutive confocal images (from dorsal to ventral) of DM3 MARCM-labeled clone (GFP, green). Secondary neurons and their projections are stained with anti-Neurotactin (Nrt, magenta). Note commissural (arrows) and longitudinal (arrowheads) projections. **(D) **Z-projection of the entire DM3 lineage. Brain and oesophagus outline shown as a magenta dashed line; midline is a white dashed line. **(E, F) **A close-up view of the commissural projections. Note how the axonal bundle defasciculates (arrows) upon entering the commissure and that a subset of these axons enters the contralateral brain hemisphere. Also note the site where DM3 axons enter the commissure (asterisk).

The DM3 lineage initial secondary axon tract split into three fiber bundles upon entering the neuropile (Figure 4A-D). Two of these bundles entered the commissural system at two sites; one site was the same as used by DM2 commissural fascicles (site 2) while the other was DM3-specific (site 3). Within the larval commissure, both axon bundles defasciculated into a meshwork of smaller bundles that crossed the midline and entered the contralateral hemisphere (Figure 4E, F). The third axon bundle formed a descending fascicle that projected along a pathway that differed from the two used by DM1 and DM2 descending fascicles.

**Figure 5:**
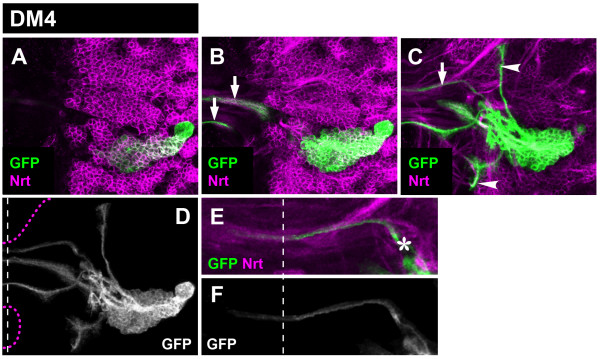
**Projection pattern of the larval DM4 lineage**. **(A-C) **Consecutive confocal images (from dorsal to ventral) of DM4 MARCM-labeled clone (GFP, green). Secondary neurons and their projections are stained with anti-Neurotactin (Nrt, magenta). Note commissural (arrows) and longitudinal (arrowheads) projections. **(D) **Z-projection of the entire DM4 lineage. Brain and oesophagus outline shown as a magenta dashed line; midline is a white dashed line. **(E, F) **A close-up view of the commissural projections. Note the site where DM4 axons enter the commissure (asterisk). Although not visible in this figure, a subset of the commissural axons enters the contralateral brain hemisphere.

The DM4 lineage had the most complex projection pattern of all the larval DM lineages. Its initial secondary axonal tract first split into three main axon bundles, and each of these formed several subsidiary axonal bundles upon entering the neuropile (Figure 5A-D). The result of this subdivision process was that the axons from this lineage formed three different and spatially separated commissural fascicles as well as one ascending fascicle and one descending fascicle. It is noteworthy that among the three commissural fascicles, only one projected in the same larval interhemispheric commissural structure as did the other DM lineages, but it did so via an entry site (site 4) different from those used by axons of the DM1-3 lineages (Figure 5E, F). The other two fascicles that projected across the midline did so in other, more dorsally located larval brain commissural structures.

**Figure 6:**
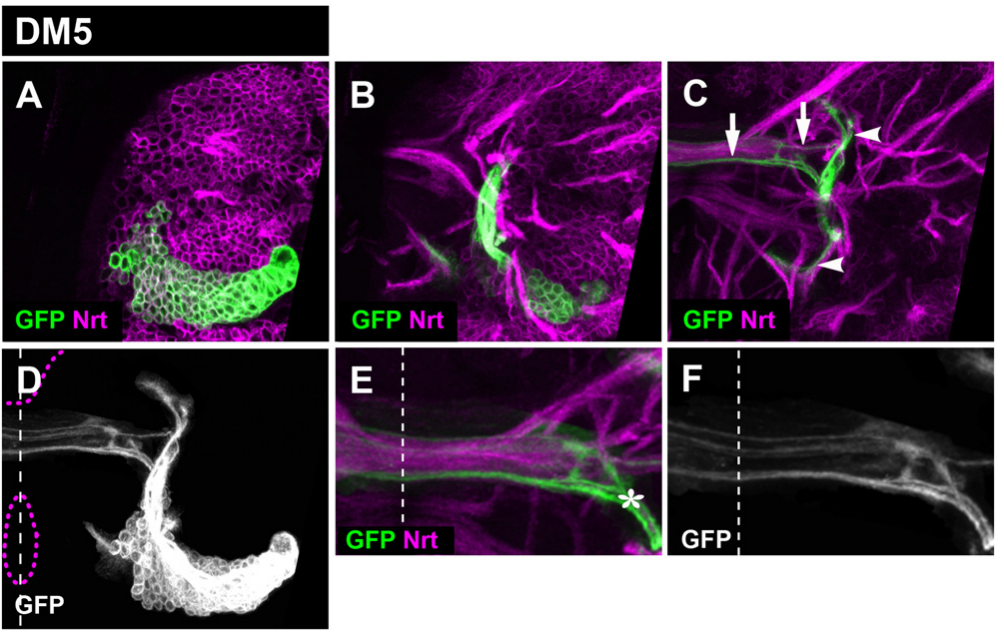
**Projection pattern of the larval DM5 lineage**. **(A-C) **Consecutive confocal images (from dorsal to ventral) of DM5 MARCM-labeled clone (GFP, green). Secondary neurons and their projections are stained with anti-Neurotactin (Nrt, magenta). Note commissural (arrows) and longitudinal (arrowheads) projections. **(D) **Z-projection of the entire DM5 lineage. Brain and oesophagus outline shown as a magenta dashed line; midline is a white dashed line. **(E, F) **A close-up view of the commissural projections. Note the site where DM5 axons enter the commissure (asterisk). Although not visible in this figure, a subset of the commissural axons enters the contralateral brain hemisphere.

The DM5 lineage secondary axon tract split into several commissural fascicles and one major ascending fiber bundle upon entering the neuropile (Figure 6A-D). The single ascending fiber bundle subdivided into two short branches. (A minor short descending fascicle was also observed; arrowheads in Figure 6B, C). All commissural fascicles of the DM5 lineage projected in the same larval commissural structure shared by the other DM lineages using the commissure entry site 4. Upon entering this commissure, the axon bundles defasciculated into smaller projections forming cross-bridging structures (Figure 6E, F).

**Figure 7:**
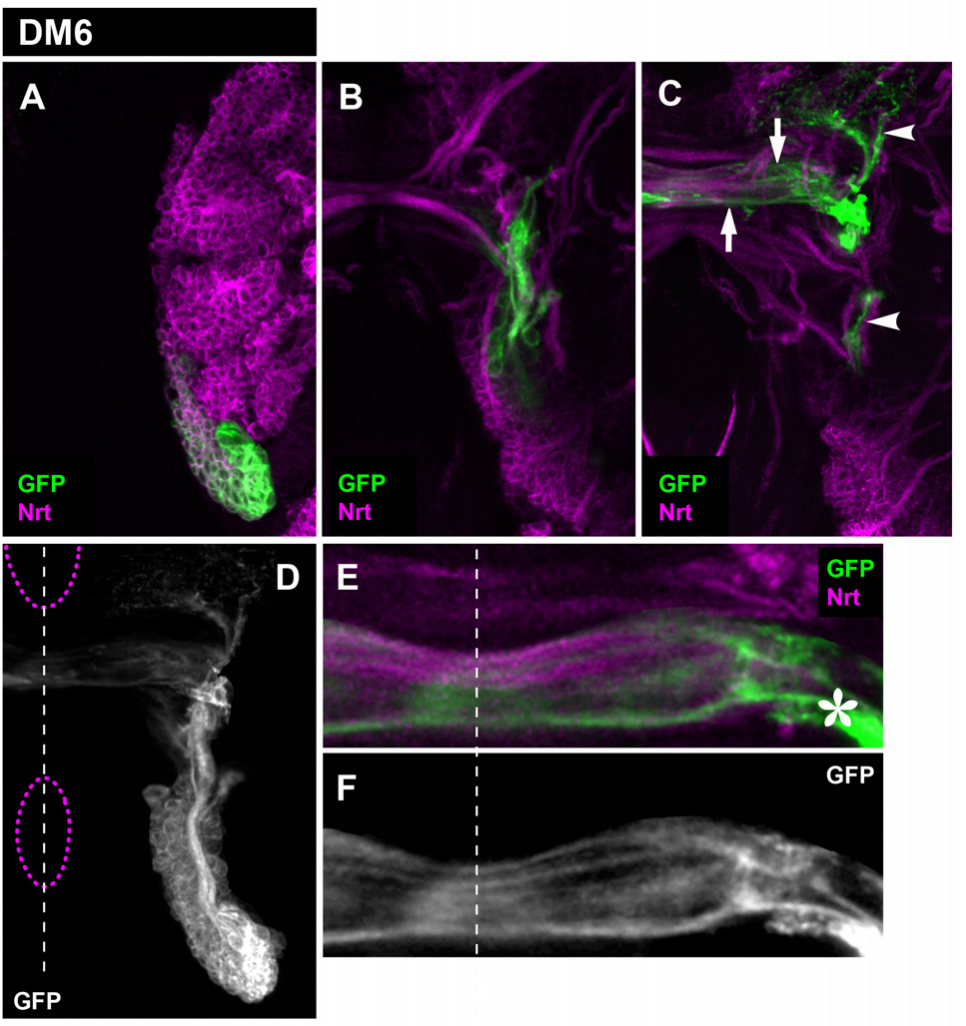
**Projection pattern of the larval DM6 lineage**. **(A-C) **Consecutive confocal images (from dorsal to ventral) of DM6 MARCM-labeled clone (GFP, green). Secondary neurons and their projections are stained with anti-Neurotactin (Nrt, magenta). Note commissural (arrows) and longitudinal (arrowheads) projections. **(D) **Z-projection of the entire DM6 lineage. Brain and oesophagus outline is shown as a magenta dashed line; midline is the white dashed line. **(E, F) **A close-up view of the commissural projections. Note the site where DM6 axons enter the commissure (asterisk). Although not visible in this figure, a subset of the commissural axons enters the contralateral brain hemisphere.

The secondary axon tract of the DM6 also split into several commissural fibers and one major ascending fiber bundle (and one minor descending bundle) upon entering the neuropile. The anatomical features of all of these fiber bundles were very similar to those of the DM5 lineage (Figure 7). Thus, commissural fascicles entered the commissure at site 4 and subsequently defasciculated, and the ascending fiber bundle split into two branches. Indeed, in comparison to the DM1-4 lineages, which are individually distinct in their axon projection pattern types, the DM5 and DM6 lineages appear to form axonal projection patterns that are largely indistinguishable.

Taken together, these findings indicate that all of the DM lineages generate highly complex secondary axon tract projections in the larval brain. Our data reveal five types of axonal projection patterns, all of which have pronounced (ipsilateral) longitudinal as well as commissural components. Four different types of axonal projection patterns can be ascribed to the DM1, DM2, DM3, and DM4 lineages and a common fifth DM5/6 type can be attributed to both DM5 and DM6 lineages.

### A Dll-Gal4 line labels DM neuroblast lineages in the postembryonic brain

**Figure 8:**
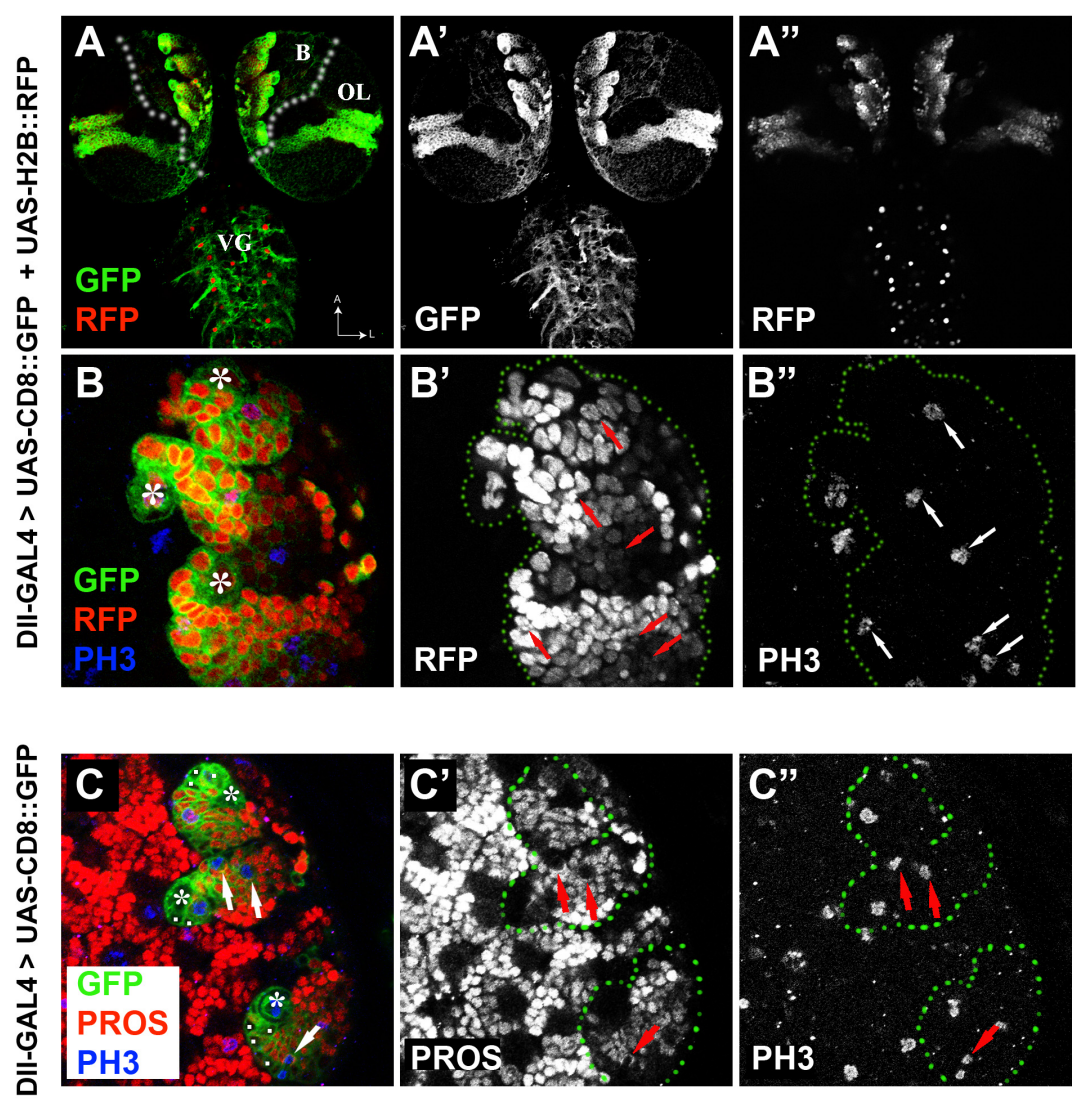
**Specific labeling of DM lineages by Dll-Gal4**. Confocal images of third instar larval nervous system expressing mCD8::GFP (green) and H2B::RFP (red in A, B) under the control of Dll-GAL4 and immunostained with anti phospho-histone 3 (PH3, blue) and anti-prospero (PROS, red in C). Panels in the left column show merged views of individual channels presented in grey scale in the middle and right columns for clarity. Green dotted lines indicate the contours of the GFP-labeled domains. **(A-A") **Dorsal view of a larval central nervous system at low magnification reveals restricted expression of the reporter genes in the medial part of the brain hemispheres (B) in the optic lobes (OL) and a few cells in the ventral ganglia (VG). **(B-B") **Higher magnification of the dorsomedial area in the left brain hemisphere. Shown are three DM neuroblasts (asterisks) and their closely associated progeny cells. Scattered cells undergoing mitosis are observed throughout the cell clusters (arrows). **(C-C") **Three DM lineages marked by Dll-GAL4 expression lack expression of Prospero in the neuroblasts and their surrounding daughter IP cells (C; asterisks and white dots, respectively). IP cells undergoing mitosis (arrows) are marked by PH3 staining (C"; arrows). They show a crescent localization of prospero (C'; arrows) in contrast to the nuclear localization of the protein in most post-mitotic cells visible in the field.

In the late larval brain, the six DM lineages located at the dorsomedial margins of each brain hemisphere can be identified based on their overall size and position by using mosaic-based MARCM techniques. In subsequent postembryonic stages, unambiguous identification of DM lineages based only on size and position is no longer possible due to the pronounced morphological changes caused by the extensive growth of neuropile in the brain and developing optic lobes. In order to identify DM lineages and analyze the cellular phenotypes of their neural cells in pupal development, we searched for Gal4 lines that might label the DM lineages. We identified such a line carrying a Gal4 transgene insertion into the promoter of the *distal-less *gene [22] (in the following referred to as Dll-Gal4), which revealed six large groups of cells at the dorsomedial margins of the brain hemisphere when coupled to the reporter genes *UAS-CD8::GFP *(membrane labeling) or *UAS-H2B::RFP *(nuclear labeling) (Figure 8A-A", B-B"). This Gal4 line also labeled cell clusters in the optic lobes as well as scattered cells in the ventral ganglia; these were not considered further here. Based on number of labeled cells as well as on their position in the developing brain hemispheres of late larval stages, the groups of cells revealed by Dll-Gal4 were likely to be DM lineages.

To confirm that the Dll-Gal4 labeled cell groups at the DM brain midline were indeed the DM lineages, a more detailed cellular and molecular analysis of the labeled cells was carried out. A characteristic hallmark of DM lineages is the fact that they contain multiple mitotically active cells located at variable distance from the parental neuroblast [12,16,17]. By using an anti-phospho-histone 3 (PH3) antibody to detect mitotic DNA (via the PH3 epitope) in larval brain expressing the reporter genes under Dll-Gal4 control, labeled cell clusters did display the expected pattern of multiple mitotic cells throughout the cluster (Figure 8B-B", C-C"). A second diagnostic feature of DM lineages is the fact that both the DM neuroblast and a few closely associated cells, the intermediate progenitors, lack nuclear Prospero expression at interphase, whereas only the intermediate progenitors typically show asymmetric cortical distribution of Prospero protein at mitosis [12,16,17]. To investigate if this was a characteristic of the Dll-Gal4 labeled cell clusters, we co-immunolabeled these cell clusters with anti-Prospero and anti-PH3 antibodies. In all cases, Dll-Gal4 labeled cell clusters had the expected immunostaining patterns. The neuroblasts and closely associated cells were Prospero-negative and mitotically active (PH3-positive), and the intermediate progenitors showed cortical Prospero crescents (Figure 8C-C"). Based on these findings, we conclude that the six cell clusters labeled by the Dll-Gal4 reporter line in the developing central brain correspond to the DM neuroblast lineages.

Taken together, these reporter line-based studies provide cellular and molecular data that confirm the identification of DM lineages based on anatomical criteria. Moreover, they provide a labeling method for DM lineages in early pupal stages.

### DM neurons form widespread arborizations and innervate the developing central complex in the pupal brain

During pupal development, the relatively simple secondary axon tracts generated by adult-specific neurons in the larval brain arborize and undergo final morphogenesis [8-11]. Given the multiplicity of longitudinal and commissural components that characterize the secondary axon tracts of the DM lineages during larval development, it might be expected that DM lineages will form multiple and widespread arborizations during pupal development. Moreover, since all six DM lineages project a subset of their fiber bundles into the larval DPC1 commissure, it seems likely that some of these arborizations will contribute to the central complex neuropile during pupal development. (The DPC1 commissure is thought to represent part of the primordium of the central complex [11].)

**Figure 9:**
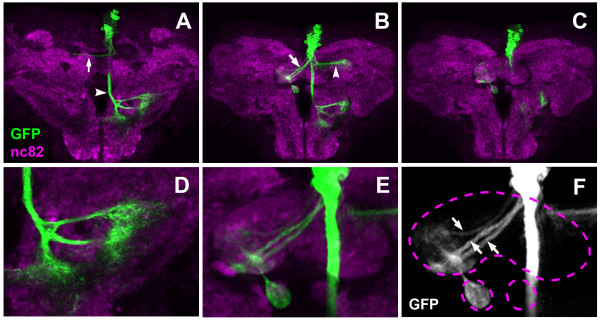
**Example of a first DM lineage projection pattern in the pupal brain 24 hours after puparium formation**. **(A-C) **Consecutive Z-projections prepared from transverse confocal sections of pupal brain neuropile. Panels show both brain hemispheres: (A) is the most dorsal, and (C) the most ventral section, with the midline in the center and anterior part pointing up (neuraxis). DM MARCM clone is labeled with anti-GFP (green); brain neuropile is labeled with anti-nc82 (magenta). Note the contralateral (arrows) and ipsilateral (arrowheads) projections. **(D) **A close-up view of the ipsilateral arborization in the posterior part of the brain. **(E, F) **A close-up view of the projections into the developing fan-shaped body and the contralateral nodulus. Note two major fascicles and one minor fascicle (arrows) entering the fan-shaped body and forming columnar arborizations. Dashed lines indicate the outlines of fan-shaped body and noduli.

**Figure 10:**
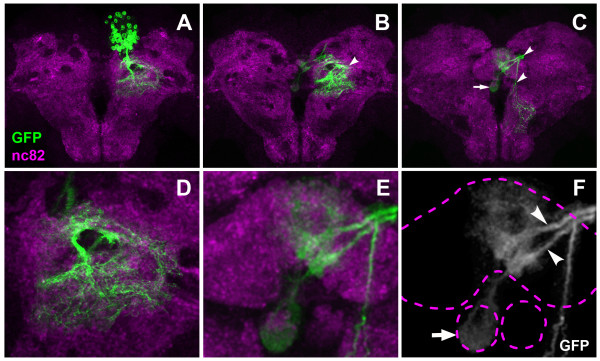
**Example of a second DM lineage projection pattern in the pupal brain 24 hours after puparium formation**. **(A-C) **Consecutive Z-projections prepared from transverse confocal sections of pupal brain neuropile. Panels show both brain hemispheres; (A) is the most dorsal, and (C) the most ventral section, with the midline in the center and anterior part pointing up (neuraxis). DM MARCM clone is labeled with anti-GFP (green); brain neuropile is labeled with anti-nc82 (magenta). Note the contralateral (arrows) and ipsilateral (arrowheads) projections. **(D) **A close-up view of the major ipsilateral arborization. **(E, F) **A close-up view of the projections into the developing fan-shaped body and contralateral nodulus. Note two fascicles (arrowheads in (F)) entering the fan-shaped body and forming columnar arborizations. Also note the arborization formed in the contralateral nodulus (arrow in (F)). Dashed lines indicate the outlines of the fan-shaped body and noduli.

To investigate this, Dll-Gal4-based MARCM labeling of DM neuroblast clones was induced at the early first instar larval stage. Labeled DM clones were recovered at 24 hours after puparium formation. (This time point for clone recovery was chosen because major neuropile structures of the future adult brain were already identifiable.) In contrast to the larval DM lineages, each of which was easily identifiable in the larval brain, the recovered MARCM-labeled pupal DM lineages could not be unambiguously individually identified since lineage-related neurons sometimes changed position and no longer formed compact groups during pupal development. In consequence, we could not assign individual arborization patterns with confidence to any one of the six pupal DM lineages. Nevertheless, two features of the adult-specific DM neurons characterized all of the MARCM-labeled DM lineages in early pupal development. First, all DM lineages formed multiple axonal projections and widespread arborizations in the brain. Second, all DM lineages projected a subset of their axon fascicles into the central complex neuropile. Two examples of the type of adult-specific arborizations formed by DM lineages in the early pupal brain are shown in Figures 9 and 10.

Figure 9 shows a MARCM-labeled DM lineage (probably a DM1 lineage) in which the cell bodies formed a discrete cluster positioned dorsally and close to the brain midline (Figure 9A-C). From this cell body cluster, a major ipsilateral descending fascicle was evident as a thick axon bundle that descended near the midline into a more ventral region of the neuropile, where it branched and formed arborizations (Figure 9A, B, D). The DM cell body cluster also gave rise to two major commissural fascicles that projected in parallel across the midline via the protocerebral bridge and entered the fan-shaped body of the central complex from the posterior aspect (Figure 9B, E, F). Within the fan-shaped body, these fascicles formed arborizations in two contralateral domains, and they also projected to and arborized in the contralateral nodulus. Moreover, they formed a restricted domain of arbors in the protocerebral bridge (data not shown). In addition to these major descending and commissural fascicles, several minor axon bundles were observed emanating from the DM neuron cell bodies; one of these formed a T-shaped bifurcation and projected ipsilaterally and contralaterally into the dorsal neuropile (Figure 9A, B).

Figure 10 shows a MARCM-labeled DM lineage (probably a DM3 lineage) in which the cell bodies formed a cluster positioned dorsally and close to the midline but located more posteriorly in the brain than the previous DM lineage (Figure 10A). From this cell body cluster, a short major axon bundle descended ipsilaterally and formed a large zone of arborizations in the dorsal brain neuropile (Figure 10A, B, D). The DM cell body cluster also gave rise to two major fascicles that projected towards the midline and entered the fan-shaped body of the central complex from the posterior aspect (Figure 10B, E, F). Within the fan-shaped body, these two fascicles formed arborizations in two ipsilateral domains, and they also projected to and arborized in the contralateral nodulus. Moreover, they formed a restricted domain of arbors in the protocerebral bridge (data not shown). In addition to these three major commissural fascicules, one minor axon bundle was observed; this descending axon bundle projected along the ipsilateral midline into posterior brain neuropile where it formed arborizations (Figure 10C).

For a more comprehensive characterization of the neuroanatomy of individual DM1-6 lineages in pupal development, lineage-specific labeling techniques will be required. For this, more selective Gal4 lines that subdivide the six DM neuroblasts into individuals will be needed. However, based on the ensemble of MARCM-labeled pupal DM lineages that were recovered and analyzed in this study, one common feature of all pupal DM lineages appears to be that a subset of the neurons in each of these lineages contributes to the developing central complex neuropile.

## Discussion

In this report we have studied the postembryonic development of the neural cells generated by the DM lineages in the larval and early pupal brain. We report three main findings. First, DM lineages comprise both adult-specific neurons and glia; DM neuroblasts thus have features of neuroglioblasts. Second, during larval development, adult-specific neurons form complex secondary axon tracts composed of separate commissural and longitudinal fascicles. Third, during pupal development the commissural fascicles arborize in and contribute to the central complex neuropile. In the following, we discuss the major implications of these three findings.

### Dorsomedial neuroblasts are multipotent neuroglial progenitors

In *Drosophila*, as in other insects, glial cells fall into three classes, surface glia, cortex glia and neuropile glia, each of which is represented in the larval brain [2]. The glial cells of the early larval brain, also referred to as primary glia, arise from a small number of embryonically active neuroglioblasts. Glial numbers in the brain increase during larval development and this increase in cell number is due, at least in part, to mitotic divisions of glial cells; however, the bulk of added (secondary) glial cells has been postulated to stem from the proliferation of unidentified secondary neuroglioblasts [23]. However, the identity of these postulated multipotent precursors in the postembryonic brain was unknown.

A recent developmental analysis by Awasaki *et al*. [21] has shown that among the different types of glial cells in the *Drosophila *brain, only the perineurial surface glia and the neuropile glia are extensively generated during postembryonic development, whereas most of the subperineurial surface glia and cortex glial cells are thought to have their origin in embryogenesis. Moreover, this analysis has provided evidence that perineurial glial precursors are distributed around the brain surface, whereas neuropile (ensheathing and astrocyte-like) glial cells are derived from specific proliferation centers within the brain. However, the progenitors of these postembryonically generated glial cells as well as the mode of postembryonic glial proliferation still remained elusive. Here we identify the first postembryonic neuroglioblasts in the *Drosophila *brain. (Embryonic neuroglioblasts have been described previously in the ventral thoracic ganglia [24].) All six DM lineages generate a set of glial cells with anatomical features of neuropile glia in addition to numerous neuronal cells during postembryonic development.

DM neuroblasts proliferate through asymmetric division that involves intermediate progenitors with transit-amplifying cell features [12,16,17]. This implies that DM lineage-derived glia, unlike any other glial cell type in *Drosophila*, are generated by amplifying intermediate progenitors. However, an exact clonal analysis of the relationship between glial cells and intermediate progenitors in DM lineages will be required to validate this notion. There is some evidence that DM derived glial are generated early in the lineage. If this is indeed the case, it is conceivable that these glial cells might be important for the differentiation of the subsequently generated neuronal cells in these lineages. For example, the extended processes of DM-derived glia located near the emergent secondary axon tracts or associated with the larval brain commissure might be important in guiding axons of DM-derived neurons (which subsequently contribute to central complex neuropile) across the midline.

### Dorsomedial lineage neurons form complex secondary axon projections

During postembryonic development, secondary, adult-specific neurons generated by reactivated neuroblasts produce secondary lineages and axons of a given secondary lineage fasciculate with each other to form a discrete and generally unbranched secondary axon tract within the brain cortex and neuropile [2,11]. In contrast, in the case of DM secondary lineages, discrete, albeit short, secondary axon tracts were visible in the cortex but were rarely observed in the neuropile. Rather, within the neuropile, the axons of any given DM lineage split into multiple axon fascicles that projected to very different parts of the brain as commissural and longitudinal axon bundles. Thus, at the anatomical level, the DM lineage already appears to be subdivided into different neuronal subgroups with different outgrowth behaviors during the larval stages.

The multiplicity of axonal bundles that emerge from a given DM lineage has features that are more reminiscent of a secondary axon tract system comprising the axon tracts of several lineages than a single secondary axon tract. This may be an indirect result of the fact that DM lineages contain three to five times more secondary neurons than do conventional neuroblast lineages, which, hence, would generate three to five times more axons than conventional lineages, resulting in an excessively large secondary axon tract in the neuropile if branching did not occur. While the underlying mechanisms are currently not known, it is also possible that this anatomical complexity is related to the particular mode of neurogenesis in the DM lineages, which involves amplifying intermediate progenitors. Thus, a given intermediate progenitor might produce neural progeny that develop a common type of axonal projection pattern, whereas progeny subsets derived from different intermediate progeny might develop different axonal projection types.

### Dorsomedial lineages contribute to the developing central complex

Like other adult-specific neurons, DM secondary neurons differentiate during the pupal period, when they evolve into the long tracts that characterize the adult brain and send out proximal as well as terminal arborizations that form synapse-rich neuropile circuitry. During the early pupal differentiation period, some of the neuropile structures that specifically characterize the adult brain become visible. Among these are the principle components of the central complex. The central complex, one of the most prominent neuropile structures in the adult brain, is located centrally between the two hemispheres and consists of four substructures. These are the protocerebral bridge, the fan-shaped body, the ellipsoid body, and the noduli, all of which are interconnected by sets of columnar interneurons that form regular patterns of projection [25-27]. A remarkable and common feature of DM lineages is that a subset of the neurons in each lineage contributes to the developing central complex neuropile.

In the late larval brain, distinguishing the four substructures of the adult central complex is difficult. However, a putative midline neuropile primordium of the central complex has been described in the late larval brain in the form of a large commissural neuropile domain, consisting of the DPC1 and trCM and probably part of the DPC2 commissures [11]. All DM lineages project a subset of their axon bundles into the DPC1 during larval development. Based on their axonal projections (and the position of their cell body clusters in the central brain), we tentatively assign the DM1-6 lineages to the DPMm1/2, DPMpm1, DPMpm2, CM4, and CM5, CM1/2 of Pereanu and Hartenstein [11]. Interestingly, in the DPC1, these commissural bundles defasciculated into smaller lattice-like projections similar to those observed in the developing central complex of the grasshopper, an insect with direct development [28].

From the first day of pupal development onward, the major components of the fan-shaped body, ellipsoid body, noduli and protocerebral bridge can be clearly recognized. In the early pupal brain, DM derived commissural neurons have already contributed to the arbors in the fan-shaped body, nodulus and protocerebral bridge. Although we were not able to identify them individually, it is very likely that the central complex-innervating DM neurons in the pupal brain are the same neurons that projected commissural axon bundles into the DPC1 commissure of the larval brain. This observation supports the notion that the DPC1 is indeed part of the central complex primordium.

A noteworthy feature of the DM-derived projections in the fan-shaped body is the fact that two major fascicles are formed that project in parallel into the fan-shaped body and form two separate arborization domains. This may reflect a contribution of a given DM lineage to a pair of the eight modular subdomains ('staves') that make up the fan-shaped body [20,25]. A similar contribution of the DM-derived neurons to two sections ('glomeruli') of the protocerebral bridge is also likely, although single cell resolution will be required to resolve this.

Most neurons of the central complex belong to one of two categories: large-field elements or small-field elements [25]. A large-field neuron typically arborizes in only a single substructure and links it to one or two central brain regions outside the central complex. Small-field neurons, as a rule, connect small columnar domains of several substructures, and the majority of small-field cells are intrinsic to the central complex. In view of the specific arborization pattern of the DM-derived neurons in the fan-shaped body, noduli and protocerebral bridge, we hypothesize that most of these neurons represent columnar small field elements of the central complex. However, it should be noted that only a subset of the neurons in any given DM lineage are likely to innervate the central complex; all DM lineages form longitudinal projections to other parts of the brain. Thus, unlike the lineages that give rise to the mushroom body intrinsic neurons, the neuronal progeny of the DM lineages are not dedicated to a single neuropile center. Rather, the unusually large number of neurons in these lineages appears to contribute to multiple, spatially separated neuropile areas in the developing brain.

## Conclusions

We have used MARCM-based clonal analysis together with immunocytochemical labeling techniques to investigate the type and fate of cells generated in the DM lineages. With a combination of neuronal and glial cell markers we show that most of the cells in the DM lineages are neuronal but that glial cells are also generated in these lineages. The DM neuroblasts thus represent the first identified precursor cells in the fly brain that have neuroglioblast functions during postembryonic development. We also show that the adult-specific neurons of each DM lineage form several spatially widely separated axonal fascicles, some of which project along larval brain commissural structures that are primordia of midline neuropile. By taking advantage of a specific Gal4 reporter line, we identify and follow DM-derived neuronal cells into early pupal stages and demonstrate that neurons of the DM lineages make a major contribution to the developing central complex, in that the numerous columnar elements are likely to be DM lineage-derived. These findings suggest that the amplification of proliferation that characterizes DM lineages may be an important requirement for generating the large number of neurons required in highly complex neuropile structures such as the central complex in the *Drosophila *brain.

## Methods

### Fly stocks and MARCM analysis

All *Drosophila *stocks were reared and maintained on standard yeast-cornmeal-agar medium and all experiments were performed at 25°C. Unless otherwise stated, fly stocks carrying transgenes and recombinant chromosomes were obtained from the Bloomington Stock Center and assembled using standard genetics. To generate positively marked MARCM clones: y, w, hsFLP; FRT40A, tubP-GAL80^LL10^/CyO, ActGFP^JMR1^; tubP-GAL4^LL7^, UAS-mCD8::GFP^LL6^/TM6, Tb, Hu were mated to w; FRT40A, UAS-mCD8::GFP^LL5 ^(standard cell lineage labeling with membrane-tethered GFP); and; FRTG13, tub-Gal80, hs-Flp/(CyO, ActGFP^JMR1^) were mated to; FRTG13, Dll-Gal4, UAS-mCD8::GFP/(CyO, ActGFP^JMR1^). The distal-less Gal4 line was; Dll-Gal4/CyO; UAS-mCD8-GFP, UAS-H2B-mRFP1.

Generation of MARCM clones and larval staging was performed as previously described [29].

### Immunohistochemistry and live imaging

Nervous systems were dissected from late third instar larvae and 24 h old pupae, and fixed and immunostained as previously described [30]. Primary antibodies were as follows: mouse anti-Repo (1:5; Developmental Study Hybridoma Bank (DSHB)), rabbit anti-Repo (1:10,000, gift from V Rodrigues), rat anti-ELAV (1:30; DSHB), mouse anti-Neurotactin (1:10; DSHB), rabbit anti-GFP (1:1,000; Molecular Probes, Eugene, Oregon, USA), mouse anti-nc82 (1:20; DSHB), mouse anti-PROS (1:10; DSHB), rabbit anti-PH3 (1:400; Upstate Biotechnology, Millipore AG, Zug, Switzerland). Alexa Fluor-conjugated secondary antibodies (Molecular Probes) were used at 1:200.

### Microscopy and image processing

Fluorescently stained nervous systems were imaged using a Leica TCS SP scanning confocal microscope using 40× and 63× oil-immersion objectives. Z stacks were collected with optical sections at 1-μm intervals. Scanned pictures were visualized in ImageJ [31] and analyzed. For the illustration of findings the most representative scans were chosen and processed (not related non-DM lineages were excluded using the 'lasso' tool). Pictures are presented as 'thick-section' merges projected as a flat image using ImageJ [31]. Figures were assembled using Adobe Illustrator and Photoshop.

## Abbreviations

DHSB: Developmental Study Hybridoma Bank; DM: dorsomedial; GFP: green fluorescent protein; MARCM: mosaic analysis with a repressible cell marker; PAN: posterior asense-negative; PH3: phospho-histone 3.

## Competing interests

The authors declare that they have no competing interests.

## Authors' contributions

NI, JB and BB carried out all the experiments. HR conceptualized the project. NI and HR wrote the manuscript.
